# Advances in vehicles for in situ delivery: From classical vectors to biologically inspired structures

**DOI:** 10.1016/j.synbio.2026.02.013

**Published:** 2026-03-17

**Authors:** Hengyi Wang, Xiaoyan Tang, Xinyao Pan, Hongjie Tang, Jie Gao, Qi Li

**Affiliations:** College of Life Sciences, Sichuan Normal University, Chengdu, 610101, China

**Keywords:** Gene delivery, Synthetic biology, Targeted delivery, Gene therapy

## Abstract

The emerging fields of synthetic biology and gene therapy rely on delivery systems to introduce the nucleic acids and proteins into recipient cells. Hence, the development of delivery tools with high specificity, strong manufacturability, and low immunogenicity can advance these fields. In this review, we summarize recent advances in the development of delivery systems for proteins and nucleic acids. First, we outline viral vector-based delivery tools, including lentivirus, adenovirus, and adeno-associated virus-based delivery technologies, discussing their advantages and limitations. Next, we summarize the advantages and disadvantages of non-viral vector-based delivery tools, including delivery strategies based on lipid nanoparticles, polyethyleneimine, exosomes, cell-penetrating peptides, virus-like particles, gold nanoparticles, and mesoporous silica nanoparticles. Lastly, we examine the specific principles and functional potential of novel delivery systems, including the Arc, PNMA2, SEND, PVC, and Coacervate systems. Overall, this review provides a systematic assessment of the mechanisms of action, current application progress, and future prospects for viral vectors, non-viral vectors, and novel delivery tools. Moreover, this review will serve as a reference for technological development and theoretical research in the fields of synthetic biology and gene therapy.

## Graphical abstract

**Figure:**
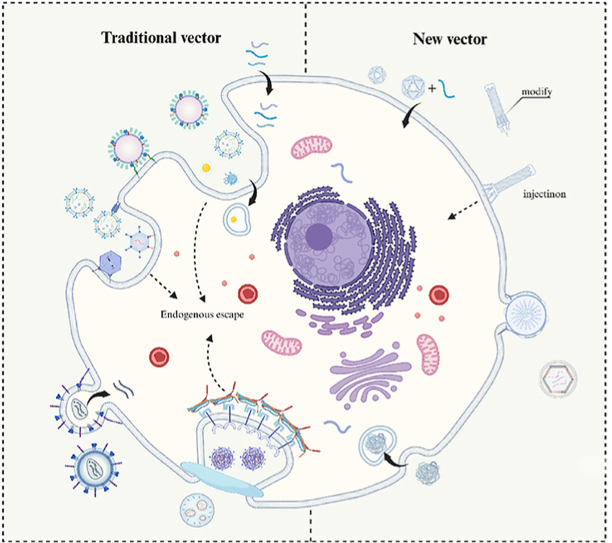

## 1 Introduction

**Fig. 1:**
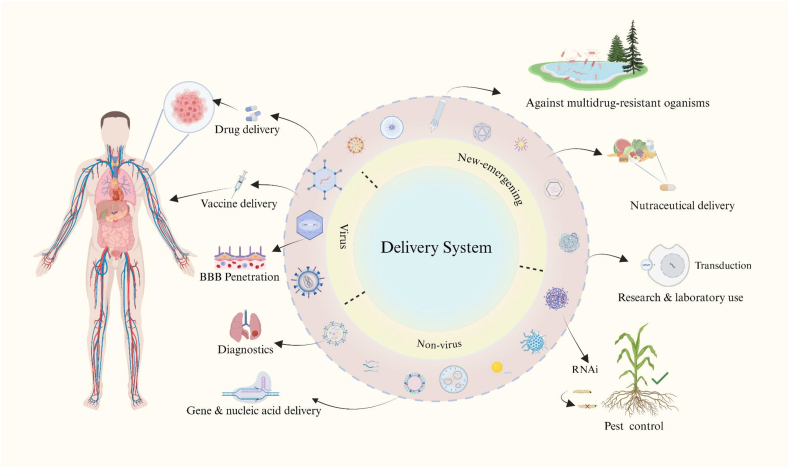
**Schematic overview of delivery systems and their diverse applications.** Delivery systems serve as versatile platforms for transporting therapeutic or functional molecules across biological barriers. Depending on their origin and design, they can be categorized into viral, non-viral, and new emerging systems. In the clinical field, they are widely used for drug and vaccine delivery, blood–brain barrier (BBB) penetration, diagnostics, and gene or nucleic acid delivery. Beyond medicine, delivery systems also find applications in agricultural pest control, nutraceutical delivery, transduction studies, laboratory research, and in combating multidrug-resistant organisms. Together, these systems illustrate the broad translational potential of delivery technologies from biomedical to environmental domains. Created in https://BioRender.com.

The emerging fields of synthetic biology and gene therapy facilitate the redesign of biological systems, and they have revolutionized the construction of functional genetic architectures [1]. The core challenge when constructing artificial biological pathways within cells or repairing pathogenic mutations is in efficiently and precisely delivering proteins/nucleic acids into target cells to achieve gene-level intervention or reprogramming. Hence, the development of delivery tools exhibiting high specificity, efficiency, and manufacturability has become a key driver for the continued advancement of synthetic biology and gene therapy. The diverse applications of delivery systems across these fields are illustrated in Fig. 1.

Since viral vectors were first used for gene transfer in 1972, viral delivery systems such as adeno-associated virus (AAV), adenovirus (AdV), and lentivirus (LV) have developed relatively mature production pipelines, laying the early foundation for molecular delivery technologies. However, because most viral vectors originate from natural structures, their high immunogenicity and limited cargo capacity continue to restrict *in vivo* applications and large-scale development. The subsequent emergence of virus-like particles (VLPs) somewhat expanded the boundaries of safety and immunological controllability. Next, non-viral delivery systems rapidly gained prominence. Lipid nanoparticles (LNPs) and polyethyleneimine (PEI) provided flexible chemical design spaces for the efficient delivery of biomacromolecules such as nucleic acids and proteins. Additionally, strategies employing inorganic nanoparticles and cell-penetrating peptides (CPPs) offered the advantages of precise targeting, structural programmability, and controlled release. However, these delivery platforms still face several challenges, including biocompatibility and *in vivo* stability. While non-viral systems typically demonstrate multiple advantageous characteristics, including scalable manufacturing, tunable chemical modification, and low immunogenicity, their transfection efficiency remains comparatively low. In recent years, a new wave of bio-inspired delivery platforms has emerged, drawing on natural biological transport mechanisms. These new platforms include endogenous loading systems based on intracellular trafficking, protein nanostructures with programmable self-assembly, self-assembling capsids derived from neuronal signaling molecules, and biomolecular condensates that mimic cellular phase separation. These systems typically demonstrate remarkable potential for enhancing targeting specificity, improving transfection efficiency, and reducing immunogenicity.

In this review, we systematically summarize the principles, applications, and respective advantages and limitations of viral vectors, non-viral vectors, and emerging bio-inspired delivery tools. By comparing these delivery strategies, we aim to elucidate the key challenges and future directions in the field. Continual evolution of these delivery technologies will help advance synthetic biology and gene therapy, and potentially inspire innovative applications in agriculture and environmental research [2,3].

## 2 Viral based delivery systems

**Fig. 2:**
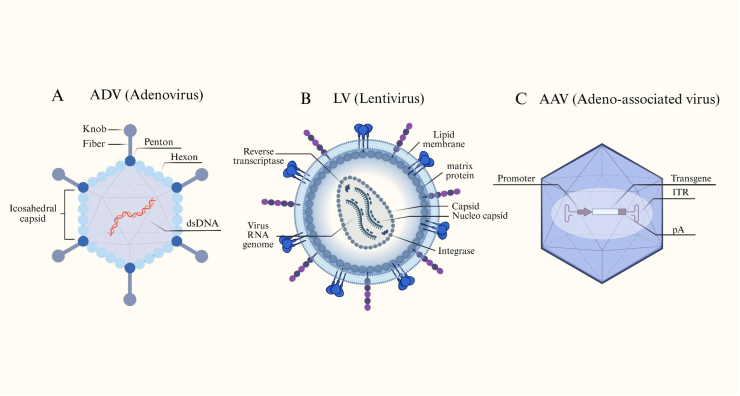
Schematic representations of major viral vector platforms used in gene delivery. (A) Adenoviral vector (AdV) featuring an icosahedral capsid composed of hexon, penton, and fiber proteins enclosing a double-stranded DNA genome. (B) Lentiviral vector (LV) derived from retroviruses, characterized by a lipid envelope containing matrix and envelope proteins, a nucleocapsid with RNA genome, and enzymes such as reverse transcriptase and integrase for genomic integration. (C) Adeno-associated virus (AAV) vector displaying a small icosahedral capsid that packages a single-stranded DNA genome flanked by inverted terminal repeats (ITRs) and driven by a promoter and polyadenylation signal for transgene expression. Created in https://BioRender.com.

Viral vectors represent the foundational delivery platforms in gene therapy, leveraging their evolutionarily optimized mechanisms for cell entry, genome protection, and efficient transgene expression. Although adeno-associated virus (AAV), adenovirus (Ad), and lentiviral (LV) vectors differ in replication strategies and cargo capacity, they share a set of defining characteristics: predictable tropism, high delivery efficiency, and extensive engineering flexibility that enables improved safety and potency. Coupled with well-established production systems, viral vectors continue to serve as the central technologies supporting both *in vivo* and *ex vivo* therapeutic applications. Fig. 2 summarizes the structural and genomic features of AdV, LV, and AAV.

### 2.1 Adenovirus: A high-capacity workhorse for gene delivery with immunogenic limitations

Adenovirus vectors (AdVs) are comprised of a non-enveloped icosahedral capsid (80–100 nm) enclosing a linear double stranded DNA genome (26–45 kb) [[4], [5], [6], [7], [8]]. Adenoviruses were identified in the 1950s as respiratory pathogens that infect epithelial cells without integrating into the host genome, and they were subsequently exploited as promising clinically validated gene delivery tools because of their advantageous attributes, which include a high transduction efficiency, a substantial cargo capacity, and the ability to remain episomal [9,10]. Notably, though AdVs are classified as a non-integrating DNA virus, adenoviral DNA can undergo rare, illegitimate integration events when delivered at high copy number like other non-integrating vectors such as AAV. In terms of their cargo capabilities, AdVs can accommodate transgenes up to 37 kb following deletion of the E3 domain, which encodes gene products that are nonessential for viral propagation [9,11]. In helper-dependent (“gutless”) variants, cargo capacity can be extended up to ∼37 kb, with these variants retaining only ITRs and packaging signals for enhanced safety, and this justifies their denomination as high-capacity AdVs (HC-AdVs) [6,12]. Because their targeted delivery range is wide, AdVs can be broadly utilized across a range of different scenarios. In total, 57 human Ad serotypes have been identified, and these often differ in their tropism. The Ad serotypes can be further sub-divided into 7 subgroups (A–G), with subgroup differences in viral capsids determining Ad serotype tropism [10]. Because AdVs can achieve extremely high transduction efficiencies in vitro and *in vivo*, they are attractive for applications requiring a quick therapeutic response. However, the proteins of all AdVs exhibit high immunogenicity [10]. Hence, pre-existing immunity to AdV proteins and acute inflammatory responses limit repeated administration of AdVs and their long-term effectiveness.

Clinically, AdVs have been successfully utilized in vaccines such as Ad26.COV2.S for COVID-19 and in oncolytic therapies like DNX-2401 [13,14]. AdVs offer cost-effective manufacturability, with production costs for adenoviral COVID-19 vaccines ranging from $0.11 to $0.23 per dose under baseline or perfusion culture conditions—potentially decreasing to $0.07 per dose with large-scale perfusion-based production—highlighting their scalability and clinical practicality [15]. However, immunological barriers remain a central limitation for AdV-based therapeutics, and consequently AdVs are fundamentally unsuitable for therapeutic applications requiring stable, long-term, or repeatable gene expression [9]. AdVs have largely been supplanted by AAV-, LNP-, and LV-based systems due to their inherent safety and performance limitations [15]. Nevertheless, AdVs will continue to play an important role in applications that capitalize on their strong transduction efficiency, large cargo capacity, and well-characterized biology, particularly in vaccine platforms and short-term gene delivery for cancer immunotherapy. A systematic comparison of viral vector–based delivery systems is provided in Table 1.

**Table 1:** Comparison of key features of AAV, adenoviral, and lentiviral vectors.

Attribute	Details	AAV (Adeno-associated virus)	ADV (Adenovirus)	LV (Lentivirus)
Structure and Biocompatibility	Molecular	Non-enveloped single-stranded DNA virus; genome∼ 4.7 kb	Non-enveloped, double-stranded DNA virus; native genome ∼26–36 kb	Enveloped, retrovirus-derived (ssRNA) vectors; genome∼8–10 kb
	Delivery Content	Primarily ∼4.7 kb ssDNA	Delivers large dsDNA transgenes; suitable for large expression cassettes	Delivers RNA genomes that are reverse transcribed and integrate for long-term expression
	Packaging capacity	<5 kb ssDNA	helper-dependent up to ∼36 kb	∼8 kb
	Modifiability	Capsid engineering; regulatory cassette tuning	Capsid (hexon/penton/fiber) and genome engineering	Envelope pseudotyping (VSV-G etc.), promoter/SIN LTRs, non-integrating variants
Delivery Activity	Delivery Efficiency	High, serotype- and tissue-dependent	Very high, transient expression	High for stable transduction of dividing and non-dividing cells, lower efficiency in myocardium tissues
	Targeting	High; efficient specific targeting of cell types such as microglia can be achieved through capsid engineering	Broad tropism; typically low specificity without retargeting modifications., requiring modifications	Moderate; can be modified by pseudotyping and envelope engineering for altered tropism
	Stability	Non-enveloped; generally physically robust and stable; capsids remain stable during low-pH transport into endosomes/lysosomes, but physical stability is also affected by temperature.	Non-enveloped; moderate physical stability but limited *in vivo* persistence due to immune responses.	Enveloped, freeze-thaw and physical stress despite pseudotyping improvements.
Safety	Immunogenicity	Generally lower innate immunogenicity than adenovirus	High innate and adaptive immunogenicity	Moderate immunogenicity *in vivo*
	Safety concerns	pre-existing NAbs; generally non-integrating.	strong innate inflammation and neutralizing Abs; limits repeat dosing; non-integrating	Insertion mutation carcinogenic risk, vector sequence recombination risk
Production	Production Difficulty	Moderate, research-scale production via HEK293 transient transfection or baculovirus/Sf9 is established	Low (easily amplified in HEK293) AdEasy has consistently been one of the most widely used AdV manufacturing systems globally	Moderate, scale-up and consistent high titre manufacturing require optimization.
	Production Cost	High per-dose manufacturing cost, driven by purification and QC for clinical-grade material.	Low, multiple simplified, efficient recombinant adenovirus construction and production systems available (e.g., AdEasy)	High production costs, primarily due to complex manufacturing processes and yield limitations
	Production Cycle	Research-scale production and purification can be completed in ∼1–2 weeks.	Rapid amplification possible (days to a few weeks depending on scale and vector type).	Typical research-scale production and concentration workflows take days to 1–2 weeks
Applications	Application Scenarios	Gene therapy for genetic disorders, development of genetically engineered animal models, and antiviral treatments	Human disease gene therapy, vaccine development (human and veterinary)	Clinical gene therapy (especially *ex vivo* engineering), animal transgenic model construction, etc
	Application Examples	Luxturna (RPE65 retinal gene replacement), onasemnogene abeparvovec (Zolgensma, systemic SMA gene therapy)	Vaccine platforms and oncolytic/anticancer therapies (Adenoviral COVID-19 vaccine platforms, oncolytic Ad agents).	*Ex-vivo* CAR-T cell therapies and HSC gene therapies
	Administration Routes	IV, intramuscular (IM), intravitreal, intracerebral, intrathecal/CSF — route depends on serotype and target tissue.	IM (vaccines), intratumoral, intranasal (some applications), IV for oncolytic approaches — systemic use limited by immune clearance.	Primarily *ex-vivo* modification of cells (e.g., T cells, HSCs)
	Advantages	Generally low pathogenicity and capable of long-term episomal transgene expression in non-dividing cells; relatively low innate immunogenicity compared with adenovirus	Non-integrating, capable of delivering large dsDNA cassettes; high innate/adaptive immunogenicity, beneficial for vaccine/oncolytic use	Efficient stable genomic integration enabling long-term expression in dividing and non-dividing cells; well-suited for *ex-vivo* cell engineering (CAR-T, HSC modification)
	Challenges	Practical payload ∼4.7 kb; pre-existing neutralizing antibodies limit systemic and repeat dosing	Strong innate and adaptive immune activation and associated inflammatory toxicity; pre-existing neutralizing antibodies limit systemic administration and repeat dosing; production requires careful QC to avoid replication-competent adenovirus or helper contamination.	Risk of insertional mutagenesis due to chromosomal integration. *In vivo* systemic delivery is constrained by envelope/serum inactivation and biosafety considerations; production scale-up requires optimization.

### 2.2 AAV: A clinically established viral vector with evolving precision and fewer limitations

Adeno-associated virus (AAV) is a clinically established gene delivery vector with a non-enveloped, icosahedral capsid (∼25 nm) enclosing a single-stranded DNA genome (∼4.7 kb) flanked by inverted terminal repeats (ITRs) [16,17]. To generate recombinant AAV (rAAV) for gene therapy, the viral rep and cap genes are first excised from the native genome, with only the ITRs being retained as cis-acting packaging signals. For gene delivery, a heterologous therapeutic expression cassette is inserted between the ITRs to create the recombinant vector. Importantly, rAAV vectors demonstrate episomal persistence with minimal genomic integration, and this major advantage is facilitated by the ITRs. As for targeting, the AAV capsid is comprised of three structural proteins (VP1:VP2:VP3 in a 1:1:10 ratio), and these are exposed at the capsid surface to determine tissue tropism [18]. Thus, characteristic changes in the amino acid sequences of the VP1–VP3 surface proteins in different serotypes determine their recognition by different cell surface receptors. In total, over 100 natural AAV serotypes (with various tissue tropisms) have already been identified, including AAV2 for the CNS, AAV8 for the liver, and AAV9 for systemic delivery [19]. Furthermore, the targeting ability of AAVs has also been expanded with engineered variants obtained through directed evolution of the capsid proteins. Notably, AAV also exhibits outstanding transduction efficiency in post-mitotic tissues such as muscle, retina, and the central nervous system, enabling long-term and stable gene expression in non-dividing cells [19,20].

Clinically, several AAV-based therapeutics have been approved by the U.S. FDA, including dalnacogene ponparvovec, etranacogene dezaparvovec, fidanacogene elaparvovec, and valoctocogene roxaparvovec, all hemophilia gene therapies designed to restore endogenous coagulation factor activity and reduce bleeding events [[21], [22], [23], [24]]. In ophthalmology, voretigene neparvovec was the first FDA-approved AAV therapy for Leber congenital amaurosis (LCA) caused by *RPE65* mutations, and this treatment demonstrates durable vision improvement. In addition, late-stage candidates such as clemidsogene lanparvovec and rebisufligene etisparvovec are advancing toward approval [25]. A list of viral vector-based gene therapy drugs is shown in Table 2.

**Table 2:** Representative virus-based therapeutics and their development status.

Drug Name	Company Name	Indication	Development Stage	Target	Mechanism of Action	
dalnacogene ponparvovec	Takeda (China) Holdings Co Ltd	Hemophilia B (Factor IX Deficiency)	Marketed	Coagulation Factor IX	Coagulation Factor IX Activator	AAV

delandistrogene moxeparvovec	Sarepta Therapeutics Inc	Duchenne Muscular Dystrophy	Marketed	Dystrophin	Dystrophin Activator	

eladocagene exuparvovec	PTC Therapeutics Inc	Aromatic l-Amino Acid Decarboxylase (AADC) Deficiency	Marketed	Aromatic L Amino Acid Decarboxylase	Aromatic L Amino Acid Decarboxylase Activator	

etranacogene dezaparvovec	CSL Behring LLC	Hemophilia B (Factor IX Deficiency)	Marketed	Coagulation Factor IX	Coagulation Factor IX Activator	

fidanacogene elaparvovec	Pfizer Inc	Hemophilia B (Factor IX Deficiency)	Marketed	Coagulation Factor IX	Coagulation Factor IX Activator	

onasemnogene abeparvovec	Novartis Healthcare A/S	Spinal Muscular Atrophy (SMA)	Marketed	Survival Motor Neuron Protein	Survival Motor Neuron Protein Activator

valoctocogene roxaparvovec	BioMarin Deutschland GmbH	Hemophilia A (Factor VIII Deficiency)	Marketed	Coagulation Factor VIII	Coagulation Factor VIII Activator

voretigene neparvovec	Novartis Pharmaceuticals Canada Inc	Leber Congenital Amaurosis (LCA); Retinitis Pigmentosa (Retinitis)	Marketed	Retinoid Isomerohydrolase	Retinoid Isomerohydrolase Activator

clemidsogene lanparvovec	RegenxBio Inc	Mucopolysaccharidosis II (MPS II) (Hunter Syndrome)	Pre-Registration	Iduronate 2 Sulfatase	Iduronate 2 Sulfatase Activator

rebisufligene etisparvovec	Ultragenyx Pharmaceutical Inc	Mucopolysaccharidosis III (MPS III) (Sanfilippo Syndrome)	Pre-Registration	N Sulphoglucosamine Sulphohydrolase	N Sulphoglucosamine Sulphohydrolase Activator

4D-150	4D Molecular Therapeutics Inc	Wet (Neovascular/Exudative) Macular Degeneration	Phase III	Placenta Growth Factor; Vascular Endothelial Growth Factor A; Vascular Endothelial Growth Factor B; Vascular Endothelial Growth Factor C	Placenta Growth Factor Inhibitor; Vascular Endothelial Growth Factor A Inhibitor; Vascular Endothelial Growth Factor B Inhibitor; Vascular Endothelial Growth Factor C Inhibitor	

AK-OTOF	Akouos Inc	Acute Sensorineural Hearing Loss	Phase III	Otoferlin	Otoferlin Activator	

arvenacogene sanparvovec	Biocad	Hemophilia B (Factor IX Deficiency)	Phase III	Coagulation Factor IX	Coagulation Factor IX Activator	

avalotcagene ontaparvovec	Ultragenyx Pharmaceutical Inc	Ornithine-Transcarbamylase Deficiency	Phase III	Ornithine Carbamoyltransferase Mitochondrial	Ornithine Carbamoyltransferase Mitochondrial Activator	

baluretgene parvec	Ocugen Inc	Retinitis Pigmentosa (Retinitis)	Phase III	Centrosomal Protein Of 290 kDa; Rhodopsin	Centrosomal Protein Of 290 kDa Activator; Rhodopsin Activator	

baluretgene parvec	Ocugen Inc	Leber Congenital Amaurosis (LCA)	Phase III	Centrosomal Protein Of 290 kDa; Rhodopsin	Centrosomal Protein Of 290 kDa Activator; Rhodopsin Activator

BBMH-803	Belief Biomed Inc	Hemophilia A (Factor VIII Deficiency)	Phase III	Coagulation Factor VIII	Coagulation Factor VIII Activator

bidridistrogene xeboparvovec	Sarepta Therapeutics Inc	Limb-Girdle Muscular Dystrophy	Phase III	Beta Sarcoglycan	Beta Sarcoglycan Activator

botaretigene sparoparvovec	Johnson & Johnson Innovative Medicine	Retinitis Pigmentosa (Retinitis)	Phase III	X Linked Retinitis Pigmentosa GTPase Regulator	X Linked Retinitis Pigmentosa GTPase Regulator Activator

Cori-Forbes Disease	Genethon SA	Glycogen Storage Disorders (GSD)	Phase III	Glycogen Debranching Enzyme	Glycogen Debranching Enzyme Activator	

esonadogene imvoparvovec	Wuhan Neurophth Biological Technology Ltd	Leber's Hereditary Optic Neuropathy (Leber Optic Atrophy)	Phase III	NADH Ubiquinone Oxidoreductase Chain 4	NADH Ubiquinone Oxidoreductase Chain 4 Activator	

GC-101	Beijing GeneCradle Technology Co Ltd	Spinal Muscular Atrophy (SMA)	Phase III	Survival Motor Neuron Protein	Survival Motor Neuron Protein Activator	

GNT-0004	Genethon SA	Duchenne Muscular Dystrophy	Phase III	Dystrophin	Dystrophin Activator	

GS-100	Grace Science LLC	Genetic Disorders	Phase III	Peptide N4, N-Acetyl Beta Glucosaminyl Asparagine Amidase	Peptide N4, N-Acetyl Beta Glucosaminyl Asparagine Amidase Activator	

GS-1191	Gritgen Therapeutics Ltd	Hemophilia A (Factor VIII Deficiency)	Phase III	Coagulation Factor VIII	Coagulation Factor VIII Activator

ixoberogene soroparvovec	Adverum Biotechnologies Inc	Wet (Neovascular/Exudative) Macular Degeneration	Phase III	Vascular Endothelial Growth Factor	Vascular Endothelial Growth Factor Inhibitor

laruparetigene zovaparvovec	Beacon Therapeutics Ltd	Retinitis Pigmentosa (Retinitis)	Phase III	X Linked Retinitis Pigmentosa GTPase Regulator	X Linked Retinitis Pigmentosa GTPase Regulator Activator

lenadogene nolparvovec	GenSight Biologics SA	Leber's Hereditary Optic Neuropathy (Leber Optic Atrophy)	Phase III	NADH Ubiquinone Oxidoreductase Chain 4	NADH Ubiquinone Oxidoreductase Chain 4 Activator

pariglasgene brecaparvovec	Ultragenyx Pharmaceutical Inc	Glycogen Storage Disease 1A	Phase III	Glucose 6 Phosphatase	Glucose 6 Phosphatase Activator

rebisufligene etisparvovec	Ultragenyx Pharmaceutical Inc	Mucopolysaccharidosis III (MPS III) (Sanfilippo Syndrome)	Phase III	N Sulphoglucosamine Sulphohydrolase	N Sulphoglucosamine Sulphohydrolase Activator	

resamirigene bilparvovec	Astellas Gene Therapies	X-Linked Myotubular Myopathy (XLMTM or MTM)	Phase III	Myotubularin	Myotubularin Activator	

RGX-202	RegenxBio Inc	Duchenne Muscular Dystrophy	Phase III	Dystrophin	Dystrophin Activator

rivunatpagene miziparvovec	Ultragenyx Pharmaceutical Inc	Wilson Disease	Phase III	Copper Transporting ATPase 2	Copper Transporting ATPase 2 Activator

sonpiretigene isteparvovec	Nanoscope Therapeutics Inc	Retinitis Pigmentosa (Retinitis)	Phase III			

SRP-9005	Sarepta Therapeutics Inc	Limb-Girdle Muscular Dystrophy	Phase III	Gamma Sarcoglycan	Gamma Sarcoglycan Activator	

surabgene lomparvovec	AbbVie Inc	Wet (Neovascular/Exudative) Macular Degeneration	Phase III	Vascular Endothelial Growth Factor A	Vascular Endothelial Growth Factor A Inhibitor	

VGNR-09	Shanghai Vitalgen BioPharma Co Ltd	Aromatic l-Amino Acid Decarboxylase (AADC) Deficiency	Phase III		

VGR-R01	Shanghai Vitalgen BioPharma Co Ltd	Retinal Degeneration	Phase III	Cytochrome P450 4V2	Cytochrome P450 4V2 Activator

ZVS-101e	Beijing Chinagene Tech Co Ltd	Retinal Degeneration	Phase III	Cytochrome P450 4V2	Cytochrome P450 4V2 Activator

Gendicine	Shenzen SiBiono GeneTech Co Ltd	Head And Neck Squamous Cell Carcinoma (HNSC); Nasopharyngeal Cancer	Marketed	Cellular Tumor Antigen p53	Cellular Tumor Antigen p53 Activator	AD

nadofaragene firadenovec	Ferring Pharmaceuticals Inc	Non Muscle Invasive Bladder Cancer (NMIBC) (Superficial Bladder Cancer)	Marketed	Interferon Alpha/Beta Receptor 2	Interferon Alpha/Beta Receptor 2 Activator	

aglatimagene besadenovec	Candel Therapeutics Inc	Prostate Cancer	Phase III	DNA Polymerase	DNA Polymerase Inhibitor	

E−10A	Marsala Biotech Inc	Recurrent Head And Neck Squamous Cell Carcinoma	Phase III			

Gene Therapy for High-Grade Glioma and Liver Transplantation	Huazhong University of Science & Technology	Liver Transplant Rejection	Phase III			

aglatimagene besadenovec	Candel Therapeutics Inc	Anaplastic Astrocytoma; Recurrent Glioblastoma Multiforme (GBM)	Phase II	DNA Polymerase	DNA Polymerase Inhibitor	

aglatimagene besadenovec	Candel Therapeutics Inc	Non-Small Cell Lung Cancer	Phase II	DNA Polymerase	DNA Polymerase Inhibitor

aglatimagene besadenovec	Candel Therapeutics Inc	Pancreatic Ductal Adenocarcinoma	Phase II	DNA Polymerase	DNA Polymerase Inhibitor

EG-011	Ferring Ventures Ltd	Refractory Angina	Phase II	Vascular Endothelial Growth Factor D	Vascular Endothelial Growth Factor D Activator

encoberminogene rezmadenovec	XyloCor Therapeutics Inc	Coronary Artery Disease (CAD) (Ischemic Heart Disease)	Phase II	Vascular Endothelial Growth Factor	Vascular Endothelial Growth Factor Activator

encoberminogene rezmadenovec	XyloCor Therapeutics Inc	Refractory Angina	Phase II	Vascular Endothelial Growth Factor	Vascular Endothelial Growth Factor Activator

enekinragene inzadenovec	Pacira BioSciences Inc	Osteoarthritis	Phase II	Interleukin 1 Receptor	Interleukin 1 Receptor Antagonist

gedeptin	GeoVax Labs Inc	Nasopharyngeal Cancer; Recurrent Head And Neck Squamous Cell Carcinoma; Salivary Gland Cancer; Solid Tumor	Phase II	Purine Nucleoside Phosphorylase	Purine Nucleoside Phosphorylase Activator	

Gene Therapy to Activate Thymidine Kinase for Prostate Cancer	The Methodist Hospital System	Prostate Cancer	Phase II	Thymidine Kinase	Thymidine Kinase Activator	

MTG-201	Momotaro-Gene Inc	Malignant Pleural Mesothelioma	Phase II			

MTG-201	Momotaro-Gene Inc	Prostate Cancer	Phase II			

RT-100	Renova Therapeutics Inc	Systolic Heart Failure (HFrEF)	Phase II	Adenylate Cyclase Type 6	Adenylate Cyclase Type 6 Activator	

RZ-001	Rznomics Inc	Astrocytoma; Hepatocellular Carcinoma	Phase II	Telomerase Reverse Transcriptase	Telomerase Reverse Transcriptase Inhibitor

RZ-001	Rznomics Inc	Glioblastoma Multiforme (GBM)	Phase II	Telomerase Reverse Transcriptase	Telomerase Reverse Transcriptase Inhibitor

TG-1042	Stamford Pharmaceuticals Inc	Basal Cell Carcinoma (Basal Cell Epithelioma)	Phase II	Interferon Gamma	Interferon Gamma Activator

TG-1042	Stamford Pharmaceuticals Inc	Basal Cell Carcinoma (Basal Cell Epithelioma)	Phase II	Interferon Gamma	Interferon Gamma Activator

TG-1042	Stamford Pharmaceuticals Inc	Gorlin Syndrome (Basal Cell Nevus Syndrome/Nevoid Basal Cell Carcinoma Syndrome)	Phase II	Interferon Gamma	Interferon Gamma Activator

BD-111	Shanghai BDgene Therapeutics Co Ltd	Simplexvirus (HSV) Infections	Phase II			LV

BD-111	Shanghai BDgene Therapeutics Co Ltd	Viral Keratitis	Phase II			

BD-113	Shanghai BDgene Therapeutics Co Ltd	Open-Angle Glaucoma	Phase II	Myocilin	Myocilin Inhibitor	

BI-3720931	Boehringer Ingelheim International GmbH	Cystic Fibrosis	Phase II	Cystic Fibrosis Transmembrane Conductance Regulator	Cystic Fibrosis Transmembrane Conductance Regulator Activator

Gene Therapy to Activate ABCD1 for Adrenoleukodystrophy	Shenzhen Geno-Immune Medical Institute	Adrenoleukodystrophy (X-Linked Adrenoleukodystrophy (X-ALD))	Phase II	ATP Binding Cassette Sub Family D Member 1	ATP Binding Cassette Sub Family D Member 1 Activator

Despite these successes, the upper practical limit for packable foreign gene fragments is ∼4.5 kb; for larger therapeutic genes, truncation of promoters or the adoption of split-vector systems (with each component encapsulated in a separate rAAV capsid) are two common strategies used to overcome this hurdle [10,19]. Additionally, because 30–60% of the population present with pre-existing neutralizing antibodies (NAbs) and may develop an immune response, there are limitations on using AAV for repeat administration, which is a significant clinical barrier [26]. Approaches to circumvent this immunity include immune-modulatory protocols (such as co-administration of rituximab), and the development of alternative capsids. Thus, changing the AAV serotype may bypass the pre-existing NAbs to enable secondary dosing [27].

Recent developments in this field have focused on dual-vector trans-splicing systems to overcome payload limits, structure-guided capsid engineering for CNS penetration (e.g., blood-brain barrier crossing AAV-PHP.eB), and hybrid designs combining AAV with lipid nanoparticles to evade immune clearance [[28], [29], [30], [31], [32]]. In 2015, Junghae Suh and colleagues incorporated a “stimuli-responsive switch” into the AAV vector to achieve spatiotemporal precise regulation, facilitating switchable nuclear translocation through a VP2-PIF6 fusion capsid combined with PhyB-NLS cellular expression [33]. In 2021, Martin Fussenegger and colleagues further improved targeting specificity by modifying the surface sequence/structure of AAV capsid proteins (VP1/VP2/VP3)—for example, an integrin-binding peptide was inserted into the VR VIII loop (position 587) of AAV2 [34]. In 2025, to overcome the challenges associated with AAV production for gene therapy, Michael Delahaye and colleagues exploited the closed, semi-automated “Quantum Hollow Fiber bioreactor” for AAV production [35]. That same year, Dan Wang and colleagues used AAVPure^Mfg^ Technology to enhance AAV purity, ensuring that the vector packaged only therapeutic genes (without generating empty capsids) [36]. Despite these considerable advances in the AAV delivery platform, challenges persist in manufacturing scalability (especially yield variability in triple-plasmid systems) and long-term expression durability in dividing cells [19].

AAVs, currently the most widely used viral vector, will likely remain the dominant “go-to” vector for *in vivo* gene therapies targeting small size genes. Its continued strength is most likely to remain in monogenic diseases and localized applications. Considering the risk of occasional genomic integration, AAV's reign may gradually give way to novel delivery systems. Horizontal comparison demonstrates the risk of viral vector genomic integration. Adenoviral DNA can undergo rare, illegitimate integration events when delivered at high copy number. For context, high-capacity adenoviral vectors (HC-AdVs) show a heterologous integration frequency of ∼6.72 × 10^−5^ events per hepatocyte. In comparison, rAAV vectors generally display integration frequencies in the range of 10^−4^–10^−6^ ISs/cell, depending on dose, serotype, and detection methodology [37,38].

### 2.3 Lentiviral vectors: integrating platforms for durable gene expression and challenges

Lentiviral vectors (LVs), primarily derived from HIV-1, are characterized by an enveloped architecture (consisting of a cylindrical or conical core) encapsulating a single-stranded RNA genome (∼8–9 kb) that is reverse transcribed into integration-competent DNA [[39], [40], [41]]. LVs typically feature a minimal set of capsid proteins, pseudotyped envelope glycoproteins (usually VSV-G), a cargo capacity of up to 9 kb, LTRs as essential cis-acting elements, and a ψ packaging signal for viral assembly [42]. Uniquely, LVs can efficiently transduce both dividing and non-dividing cells via receptor-mediated endocytosis, achieving stable genomic integration [43].

Clinically, several LV-based treatments have already been approved by the U.S. FDA, including landmark therapeutics such as Zynteglo for β-thalassemia and Strimvelis for ADA-SCID, while next-generation vectors containing mutated capsid proteins for reduced immunogenicity and including suicide gene switches for enhanced controllability are currently being evaluated in trials [44,45]. These successes notwithstanding, *in vivo* applications of LVs continue to face significant challenges, including innate immune recognition via TLR-mediated pathways and unintended accumulation in the liver and spleen [[46], [47], [48]]. However, these limitations can be mitigated through strategies such as PEGylation or the use of myeloid-specific miRNA decoy systems [49,50]. The targeting potential of LVs can be further enhanced using envelope engineering. For example, by incorporating measles glycoproteins to confer lymphocyte specificity, through the use of synthetic promoters derived from CD34 to restrict expression to hematopoietic lineages, and by using self-inactivating (SIN) LTR designs and targeted integration systems to address safety concerns about insertional mutagenesis [51,52]. Persistent bottlenecks include manufacturing scalability, which is constrained by low yields from adherent production platforms, and in ensuring vector stability during systemic administration [53]. Ongoing innovations—such as stable producer cell lines and optimized lyophilization protocols—are designed to address these limitations [54].

Lentiviral vectors will remain the dominant platform for CAR-T and other autologous cell therapies, but their role is shifting. Rather than serving as broad gene-delivery vehicles, they are increasingly positioned as specialized tools for durable *ex vivo* cellular reprogramming, while next-generation *in vivo* gene therapies will likely be driven by non-integrating or non-viral delivery modalities.

## 3 Non-viral delivery systems in gene therapy

**Fig. 3:**
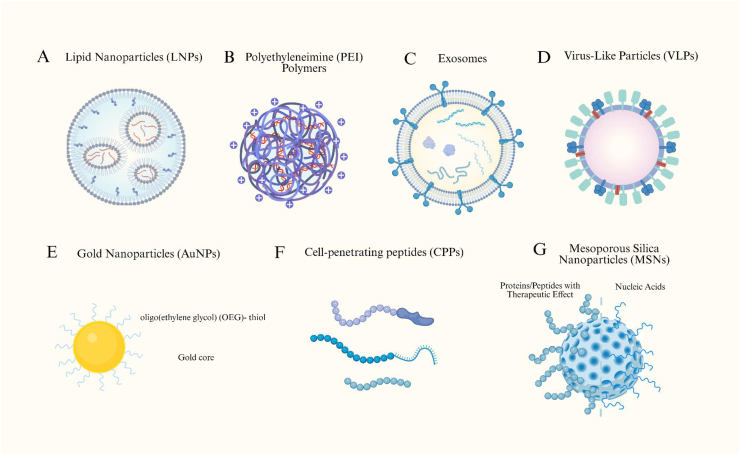
Representative types of non-viral vectors for biomolecular delivery. Non-viral vectors offer versatile and safer alternatives to viral systems for the transport of nucleic acids, proteins, and small molecules. Common platforms include (A) lipid nanoparticles (LNPs) for mRNA and siRNA delivery; (B) polyethyleneimine (PEI) polymers that form electrostatic complexes with nucleic acids; (C) exosomes (extracellular vesicles) that utilize endogenous transport pathways; (D) virus-like particles (VLPs) mimicking viral structures without genetic material. (E) Gold nanoparticles (AuNPs). (F) Cell-penetrating peptides (CPPs). (G) mesoporous silica nanoparticles (MSNs) enable tunable surface modification, controlled release, and targeted intracellular delivery. Created in https://BioRender.com.

Non-viral delivery systems have emerged as versatile and increasingly clinically validated tools for gene therapy, offering improved safety profiles, scalable manufacturing, and broad payload compatibility compared with traditional viral vectors. These systems enable efficient encapsulation, protection, and intracellular delivery of nucleic acids, proteins, and genome-editing cargos. Mechanistically, they mediate cellular entry through endocytosis or membrane interaction, followed by endosomal escape and cytosolic payload release via lipid fusion, or direct membrane destabilization. Rapid advances in chemical design, modular engineering, and targeted surface functionalization continue to expand the therapeutic landscape of non-viral vectors. Fig. 3 summarizes the structure of non-viral delivery systems.

### 3.1 Lipid nanoparticles (LNPs) as vectors in ex vivo gene therapy

Lipidoids were first demonstrated in 2008 to achieve highly efficient delivery of RNA interference *in vivo* [55]. Advances between 2012 and 2016 optimized ionizable lipid chemistry—particularly the introduction of pKa-tuned amino lipids—which dramatically improved potency and tolerability for systemic mRNA and siRNA delivery [56]. These developments converged into the modern lipid nanoparticles (LNPs). LNPs are non-viral delivery systems with a molecular architecture characterized by a 100 nm vesicular structure composed of four core components: ionizable cationic lipids, cholesterol, helper phospholipids (DSPC or DOPE), and PEGylated lipids [57]. This molecular architecture enables efficient encapsulation and protection of diverse nucleic acid payloads, including mRNA and siRNA, shielding them from enzymatic degradation during systemic circulation [58,59]. Functionally, LNPs enter cells via receptor-mediated endocytosis; once within the acidic endosomal environment (pH < 6.0), ionizable lipids within the LNPs become protonated, promoting membrane fusion and endosomal escape, ultimately facilitating cytosolic release of the therapeutic cargo [57,60]. LNPs demonstrate significant advantages, including good biocompatibility, adjustable physicochemical properties, a high nucleic acid loading capacity, and effective protection against enzymatic degradation [61]. Recent progress highlights that further tuning ionizable lipid composition and LNP microstructure remains essential for enhancing endosomal escape and maximizing RNA delivery efficiency [62]. A list of gene therapy drugs delivered using a non-viral vector is shown in Table 4.

**Table 3:** Comparison of key features of non-virus-based delivery systems.

Attribute	Attribute	LNPs (Lipid Nanoparticles)	PEI Polymers	VLP (Virus-Like Particles)	Exosomes	CPPs (Cell-Penetrating Peptides)	Nanoparticles (AuNPs)	Nanoparticles (MSNs)
Structure and Biocompatibility	Molecular	Nanoscale spherical or polyhedral particles self-assembled from ionizable lipids, phospholipids, cholesterol, and PEG-lipids	Cationic polymers composed of repeating amine groups with tunable branching and molecular weights	Nanoparticles composed of viral structural proteins (without viral genetic material)	Naturally occurring lipid bilayer vesicles (30-150 nm) secreted by cells	Short amphipathic peptides consisting of 5-30 amino acids	Spherical metallic nanoparticles ranging from 1 to 150 nm with tunable surface chemistry	Mesoporous silica nanoparticles with high specific surface area and regular pores
	Delivery Content	mRNA, siRNA	pDNA, siRNA, mRNA	Antigens, nucleic acids, RNPs, small-molecule drugs	mRNAs, miRNAs, proteins, and small molecule drugs	Cas9 gRNA, siRNA, mRNA, small-molecule drugs	siRNA, mRNA, RNPs, pDNA, antibodies, and imaging agents	Antibiotics, antitumor drugs, siRNA
	Packaging capacity	Moderate; suitable for short to long RNA cargos	High; can efficiently compress kb-scale plasmid DNA via electrostatic interaction to form stable nanocomplexes	Low, but loading capacity can be enhanced	Low	Moderate; can bind to cargos via covalent or non-covalent methods	High; loading capacity depends on particle size, shape, and surface functionalization	High; loading capacity is precisely tunable by pore size and surface functionalization

	Details	LNPs (Lipid Nanoparticles)	PEI Polymers	VLP (Virus-Like Particles)	Exosomes	CPPs (Cell-Penetrating Peptides)	Nanoparticles (AuNPs)	Nanoparticles (MSNs)
Delivery Activity	Modifiability	Very high, tunable via ionizable lipid design, PEG-lipid ratio, lipid composition, and addition of targeting ligands; formulation critically affects biodistribution and stability.	Good, modifiable by PEGylation, ligand conjugation, or copolymerization	High, capsids can be modified via genetic engineering or chemical modification	Good, achievable via parental cell engineering, membrane bioconjugation, or fusion protein display (e.g., VSV-G)	High, sequence design, chemical conjugation, or multimer assembly can tune properties	Highly controllable in size, shape, and surface chemistry; conjugation enables functional versatility.	High, pore size, surface groups, and particle size adjustable; allows precise control of loading and release; coatings improve biocompatibility.
	Delivery Efficiency	High, efficient uptake and endosomal escape depend on formulation; ligand targeted systems can reach extrahepatic tissues.	High, exhibits "proton sponge effect"	High; but antigen stability and function may be affected by extreme pH environments	Variable, efficient in certain models, but overall limited by low natural cargo loading and artificial encapsulation efficiency.	High cellular uptake *in vitro*	High, uptake when surface-functionalized internalization mainly via endocytosis	High, uptake primarily via endocytosis
	Targeting	Passive liver tropism driven by ApoE adsorption; specific targeting requires ligand conjugation.	Poor intrinsic targeting, non-specific distribution; targeting achievable only with ligand modification.	Variable, certain VLPs have natural receptor tropism, others require engineering for targeting specificity.	Source-dependent tropism, some exosomes show tissue preference or BBB crossing	No intrinsic organ targeting, broad cellular entry, but limited tissue selectivity.	No intrinsic targeting	No intrinsic targeting
Safety	Stability	Good in vitro; *in vivo* stability depends on PEG-lipid content, ionizable lipid composition, and serum protein interactions (e.g., ApoE).	Poor in serum (easy aggregation, opsonization); limited circulation stability. PEGylation or copolymer modification improves it.	Good in vitro stability under appropriate conditions; *in vivo* stability varies by type	High stability in biological fluids.	Low; easily degraded by proteases.	Excellent in vitro physicochemical stability; *in vivo* stability depends on surface modification	Excellent in vitro chemical stability; *in vivo* stability affected by surface modification
	Immunogenicity	Low, ionizable lipids and PEG can trigger innate responses (TLR, inflammasome) and anti-PEG antibodies.	Variable; low adaptive immunogenicity but may cause inflammation and complement activation; depends on MW and branching.	High; can effectively stimulate humoral and cellular immune responses	Extremely low; benefits from endogenous origin	Low; depends on sequence and dose—long or foreign peptides may elicit immune responses.	Low; biocompatibility and potential cytotoxicity highly depend on size, shape, and surface chemistry	Low; immunological properties have a structure-activity relationship
	Safety concerns	Dose-dependent inflammation and potential hepatotoxicity due to ionizable lipids.	Dose-dependent cytotoxicity caused by high positive charge density	Generally safe (no genome), but residual host contaminants and strong immunogenicity require control.	High biosafety (no viral genome)	High doses can disrupt membranes and cause cytotoxicity or hemolysis; off-target uptake possible.	Size and surface property dependent cytotoxicity; potential risks from *in vivo* accumulation	Size- and surface property-dependent cytotoxicity; potential inflammatory responses from *in vivo* accumulation
Production	Production Difficulty	Moderate; Microfluidic formulation and lipid synthesis are well established	Low; mass-producible via mature chemical synthesis methods	High. (Recombinant protein expression, complex purification, controlled assembly, and sterility/consistency demands).	High; main bottlenecks include large-scale culture, isolation/purification, and standardization	Low; Solid-phase peptide synthesis (SPPS) is mature	Moderate	Moderate
	Production Cost	Relatively high but decreasing with scale.	Low; raw materials easily accessible, synthesis process mature and stable	Relatively high; mainly due to complex biological expression systems and downstream purification processes	High; mainly due to high costs of large-scale cell culture, complex downstream separation/purification, and low yield	Low; simple synthesis, raw materials easily available	Moderate; raw material costs controllable.	Moderate; raw material costs low.
	Advantages	Scalable and standardizable production; versatile formulation and surface functionalization for targeting.	High nucleic-acid condensation and transfection potency; chemical modifiability.	Strong cell entry and potent.	Low intrinsic immunogenicity when autologous; natural ability for intercellular transfer.	Efficient membrane translocation and adaptable design, great potential as a drug carrier for CNS diseases	Highly tuneable surface chemistry and size; excellent physical stability *in vitro*.	Very high loading capacity and controlled release via tunable pore structure
	Challenges	Passive liver tropism; potential dose dependent inflammatory responses and hepatotoxicity	Marked cytotoxicity at high MW/branched architectures; serum instability and clearance issues.	Complex and costly manufacturing; strong immunogenicity may limit repeated dosing; loading capacity.	Major scale-up and standardization bottlenecks	Limited endosomal escape for many cargos, proteolytic instability in serum, and lack of intrinsic tissue specificity.	Potential for non-specific biodistribution.	Potential biocompatibility/toxicity issues if uncoated, uncertain long-term biodegradation.
	Application	Approved COVID-19 mRNA vaccines, therapeutic siRNA drug Onpattro, etc.	Delivery of mRNA via graphene oxide-PEI complexes to generate human induced pluripotent stem cells (iPSCs)	Quadrivalent HPV VLP vaccine	Exosome therapy technologies (e.g., CAP-2003)	Use of TAT and Penetratin peptides in nose-brain delivery systems (mediate Cas9 proteins or neuroprotective proteins to the brain for gene editing or disease treatment)	Doxorubicin-loaded gold nanoparticles (delivered to tumors via active/passive targeting to reduce systemic toxicity)	Chlorotoxin-functionalized mesoporous silica nanoparticles (for pH-responsive delivery of paclitaxel to glioblastoma)
	Administration Routes	Intramuscular administration, intravenous administration, subcutaneous administration	Intramuscular administration, intravenous administration, subcutaneous administration	Intravenous injection, oral administration, respiratory inhalation (to induce systemic or mucosal immunity)	Intravenous route, subcutaneous route, intramuscular route	Intranasal administration (enables efficient nose-brain delivery)	Mainly intravenous injection and local injection/topical application (currently in early clinical trials)	Mainly intravenous injection and oral administration (currently in the "excipient industrial application" stage)

**Table 4:** Representative non-virus-based therapeutics and their development status.

Drug Name	Company Name	Indication	Development Stage	Target	Mechanism of Action	
NTLA-2002	Intellia Therapeutics Inc	Hereditary Angioedema (HAE) (C1 Esterase Inhibitor [C1–INH] Deficiency)	Phase III	Plasma Kallikrein	Plasma Kallikrein Inhibitor	LNP
ABO-101	Arbor Biotechnologies Inc	Primary Hyperoxaluria Type I	Phase II	2 Hydroxyacid Oxidase 1	2 Hydroxyacid Oxidase 1 Inhibitor	
ABOD-2011	Suzhou Abogen Biosciences Co Ltd	Head And Neck Squamous Cell Carcinoma (HNSC); Melanoma; Solid Tumor	Phase II	Interleukin 12	Interleukin 12 Activator	
ABOD-2011	Suzhou Abogen Biosciences Co Ltd	Hepatocellular Carcinoma	Phase II	Interleukin 12	Interleukin 12 Activator	
ARCT-032	Arcturus Therapeutics Holdings Inc	Cystic Fibrosis	Phase II	Cystic Fibrosis Transmembrane Conductance Regulator	Cystic Fibrosis Transmembrane Conductance Regulator Activator	
ARCT-810	Arcturus Therapeutics Holdings Inc	Ornithine-Transcarbamylase Deficiency	Phase II	Ornithine Carbamoyltransferase Mitochondrial	Ornithine Carbamoyltransferase Mitochondrial Activator	
BEAM-301	Beam Therapeutics Inc	Glycogen Storage Disease 1A	Phase II	Glucose 6 Phosphatase	Glucose 6 Phosphatase Activator	
BEAM-302	Beam Therapeutics Inc	Alpha-1 Antitrypsin Deficiency (A1AD)	Phase II	Alpha 1 Antitrypsin	Alpha 1 Antitrypsin Activator	
BNT-142	BioNTech SE	Endometrial Cancer; Fallopian Tube Cancer; Metastatic Ovarian Cancer; Non-Small Cell Lung Cancer; Peritoneal Cancer; Solid Tumor; Testicular Cancer	Phase II	CD3; Claudin 6	CD3 Agonist; Claudin 6 Inhibitor	
CTX-310	CRISPR Therapeutics AG	Heterozygous familial hypercholesterolemia (heFH); Homozygous Familial Hypercholesterolemia (HoFH); Hypertriglyceridemia; Mixed Dyslipidemia	Phase II	Angiopoietin Related Protein 3	Angiopoietin Related Protein 3 Inhibitor	
ETH-47	ethris GmbH	Asthma	Phase II	Type III Interferon		
HN-2301	Shenzhen MagicRNA Biotech Co Ltd	Myasthenia Gravis	Phase II	B Lymphocyte Antigen CD19	B Lymphocyte Antigen CD19 Inhibitor	
HN-2301	Shenzhen MagicRNA Biotech Co Ltd	Systemic Lupus Erythematosus	Phase II	B Lymphocyte Antigen CD19	B Lymphocyte Antigen CD19 Inhibitor	
mRNA-3705	Moderna Inc	Methylmalonic Acidemia	Phase II	Methylmalonyl CoA Mutase Mitochondrial	Methylmalonyl CoA Mutase Mitochondrial Activator	
mRNA-3745	Moderna Inc	Glycogen Storage Disease 1A	Phase II	Glucose 6 Phosphatase	Glucose 6 Phosphatase Activator	
mRNA-3927	Moderna Inc	Propionic Acidemia	Phase II	Propionyl CoA Carboxylase Alpha Chain Mitochondrial; Propionyl CoA Carboxylase Beta Chain Mitochondrial	Propionyl CoA Carboxylase Alpha Chain Mitochondrial Activator; Propionyl CoA Carboxylase Beta Chain Mitochondrial Activator	
PBGENE-HBV	Precision Biosciences Inc	Hepatitis B	Phase II	cccDNA	cccDNA Inhibitor	
RCT-2100	Recode Therapeutics Inc	Cystic Fibrosis	Phase II	Cystic Fibrosis Transmembrane Conductance Regulator	Cystic Fibrosis Transmembrane Conductance Regulator Activator	nanoparticles
RXRG-001	RiboX (Shanghai) Therapeutics Co Ltd	Xerostomia	Phase II	Aquaporin 1	Aquaporin 1 Activator	
STX-001	Strand Therapeutics Inc	Melanoma; Solid Tumor; Triple-Negative Breast Cancer (TNBC)	Phase II	Interleukin 12	Interleukin 12 Activator	
YOLT-201	YolTech Therapeutics Co Ltd	Familial Amyloid Cardiomyopathy; Familial Amyloid Neuropathies	Phase II	Transthyretin	Transthyretin Inhibitor	
IMNN-001	Imunon Inc	Epithelial Ovarian Cancer; Fallopian Tube Cancer; Peritoneal Cancer	Phase III	Interleukin 12 Subunit Alpha; Interleukin 12 Subunit Beta	Interleukin 12 Subunit Alpha Activator; Interleukin 12 Subunit Beta Activator	
IMNN-001	Imunon Inc	Adenocarcinoma; Endometrial Cancer; Epithelial Tumor	Phase II	Interleukin 12 Subunit Alpha; Interleukin 12 Subunit Beta	Interleukin 12 Subunit Alpha Activator; Interleukin 12 Subunit Beta Activator	
quaratusugene ozeplasmid	Genprex Inc	Small-Cell Lung Cancer	Phase II	GATOR Complex Protein NPRL2; Tumor Suppressor Candidate 2	GATOR Complex Protein NPRL2 Activator; Tumor Suppressor Candidate 2 Activator	
quaratusugene ozeplasmid	Genprex Inc	Non-Small Cell Lung Cancer	Phase II	GATOR Complex Protein NPRL2; Tumor Suppressor Candidate 2	GATOR Complex Protein NPRL2 Activator; Tumor Suppressor Candidate 2 Activator	

Features mentioned above underpinned the clinical success of COVID-19 mRNA vaccines [57], which demonstrated exceptionally high efficiency (95% and 94.5%) [63]. LNPs manufactured using microfluidic processes cost less than $10 per dose, with throughputs of up to 3 million doses per minute [64]. In oncology, LNPs facilitate targeted delivery of chemotherapeutic agents, tumor-specific antigens for CAR-T cell therapy (e.g., marking tumors with VHH antibodies), and immunomodulatory agents such as PD-L1 inhibitors, converting immunologically "cold" tumors to "hot" tumors [65]. LNPs also enable efficient systemic delivery of CRISPR-Cas components for gene correction in preclinical models.

These considerable advantages notwithstanding, there are several intrinsic limitations of LNPs. The main challenges include their low endosomal escape efficiency, PEG-associated immunogenicity, limited *in vivo* stability, and scalability, and these problems continue to constrain the broader therapeutic applications of LNP-based systems [60,[66], [67], [68], [69]]. Recently, enrollment for the gene therapy drug VERVE-101 was paused in its Phase 1b (Heart-1) clinical trial after a participant experienced elevated liver transaminases (ALT/AST) and thrombocytopenia, two adverse events attributed to the LNP delivery shell, underscoring ongoing safety concerns in LNP-mediated gene delivery. Following this setback, Verve Therapeutics will now focus on the development of VERVE-102. LNP hepatotropism is caused by an accumulation of unmodified LNPs in the liver via ApoE-mediated uptake [70]. To address this major issue, Selective Organ Targeting (SORT) modifications have been developed, enabling redistribution of LNPs to organs beyond the liver, including the lungs, spleen, and lymph nodes [71,72].

### 3.2 Polyethyleneimine: A classical synthetic polyplex system for safer, targeted delivery

Polyethyleneimine (PEI) is a synthetic, cationic polymer capable of forming stable polyplexes with negatively charged nucleic acids via electrostatic interactions, making it a useful non-viral gene delivery vector [73,74]. Structurally, PEI exists in both linear and branched forms, with branched 25 kDa variants being particularly effective attributed to its dense cationic charge density that promotes robust binding with DNA, RNA, and plasmids, only polyplexes formed with 25 kDa PEI that exhibited small particle sizes (approximately 59–80 nm) and strongly positive zeta potentials (around +40 mV) were capable of efficiently delivering siRNA [75]. PEI enables endosomal escape through multiple cooperative mechanisms. PEI-mediated transfection relies on electrostatic adsorption to the cell membrane, endocytic uptake primarily through clathrin-mediated pathways, and with the “proton sponge effect” serving as the core mechanism for endosomal escape [75]. Sondergaard et al., highlighting their key finding confirmed via nano-pH sensors: “PEI does not significantly alter lysosomal pH, and its concentration is insufficient to trigger osmotic rupture” [76]. The classical proton sponge effect contributes but is not sufficient on its own. Current evidence shows that direct PEI–membrane interactions such as electrostatic binding, membrane thinning, and pore formation are the primary mechanism of escape. Additional contributions include lipid reorganization toward non-lamellar phases and partial lipid extraction, which further destabilize endosomal membranes [77].

In *in vitro* applications, the transfection efficiency of PEI is typically high across a diverse range of cell lines and tissues, and it demonstrates efficient nucleic acid condensation and protection abilities [78,79]. However, in *in vivo* applications, the performance of PEI gene delivery vectors is hampered by serum inhibition, instability, and high cytotoxicity (caused by its strong positive surface charge) [80,81]. Moreover, PEI lacks inherent targeting specificity, leading to nonspecific accumulation in organs such as the liver and lungs. To address these challenges, current research is focused on structural modifications that enhance the safety and efficacy of PEI gene delivery vectors. Chemical conjugation with targeting ligands (e.g., peptides) or antibodies against the RGD motif enables cell-selective uptake [82]. PEGylation has been shown to reduce immunogenicity and to prolong circulation time, while the incorporation of biodegradable linkages (e.g., ester bonds) can help mitigate long-term toxicity. Together, these engineering strategies can be used to optimize the therapeutic index of PEI for clinical applications in gene therapy and vaccine development. Polyethylenimine (PEI) will remain a workhorse for in-vitro transfection and localized nucleic acid delivery, however, its intrinsic non-biodegradability fundamentally limits its suitability as a safe, clinically viable platform for systemic *in vivo* gene therapy. By contrast, fully degradable polyester-based carriers—exemplified by BCPVs and biodegradable polymeric nanoparticles for *in vivo* siRNA delivery—demonstrate how rational polymer design can preserve transfection potency while markedly reducing long-term toxicity [83,84]. Multifunctional polymeric nanocarriers are also being engineered for combination therapy; for example, chemically cross-linked cationic PEG–PLA nanocapsules capable of co-delivering P-gp siRNA and doxorubicin have been shown to effectively reverse multidrug resistance in breast cancer cells [85].

### 3.3 Virus-like particles (VLPs): virus-inspired nanocarriers bridging synthetic and biological delivery platforms

Virus-Like Particles (VLPs) constitute a "virus-derived non-viral system" that self-assembles from viral structural proteins, but lacks viral genetic material; thus, VLPs combine the delivery efficiency of viruses with enhanced biosafety [86,87]. Structurally, VLPs are classified as either enveloped or non-enveloped, with their architecture determined by the capsid proteins of the parental virus. While enveloped VLPs achieve cytosolic release via pH-dependent membrane fusion, non-enveloped VLPs typically rely on receptor-mediated endocytosis [88]. These differences notwithstanding, enveloped and non-enveloped VLPs both facilitate the encapsulation and delivery of gene-editing complexes in the form of ribonucleoproteins (RNPs). Additionally, the cellular and tissue selectivity of VLP platforms can be modulated through judicious choice of the viral proteins incorporated into the capsid shell, thereby exploiting the diversity of known viruses and their distinct tropisms. Overall, this strategy minimizes the risks of insertional mutagenesis and host immune activation caused by viral components and sustained transgene expression [89,90].

By exploiting native viral entry mechanisms, VLPs typically achieve delivery efficiencies comparable to those of infectious viruses. Banskota et al. developed fourth-generation engineered virus-like particles (eVLPs) capable of efficiently packaging and delivering therapeutic proteins or gene-editing ribonucleoproteins *in vivo*, and achieved high editing efficiency with minimal off-target effects and without genomic integration [89]. Current applications of VLPs vary widely, spanning prophylactic and therapeutic vaccine development, targeted cancer therapies, and the delivery of gene editing therapies. Geilenkeuser et al. engineered nucleo-cytosolic vehicles (ENVLPEs) that selectively package CRISPR editors via gRNA-aptamer recognition, enabling efficient in vitro and *in vivo* genome editing in primary T cells and retinal disease mouse models [91].

Despite their many advantages, VLPs face significant challenges that limit their broader use in systemic gene therapy. They often trigger strong innate and adaptive immune responses, which can compromise repeated administration and reduce circulation time [88,92,93]. As a result, VLPs are unlikely to replace engineered viral vectors for long-term gene delivery or applications that require repeated systemic dosing. Instead, they are most likely to continue dominating fields where high immunogenicity, multivalent display, and precise antigen presentation are critical. Ongoing efforts to overcome the intrinsic limitations of VLPs are focused on optimizing expression systems and applying surface engineering strategies such as PEGylation to enhance pharmacokinetic behavior and minimize immune recognition [94]. As mentioned above, the targeting specificity of VLPs is primarily determined by the viral proteins incorporated into the capsid shell. By employing engineering approaches such as pseudotyping—using heterologous envelope proteins like VSV-G to broaden tropism—and the redesign of surface glycoproteins, researchers aim to achieve precise and controllable cell-type targeting [95,96].

### 3.4 Exosomes (extracellular vesicles): endogenous vesicular systems for clinical-grade targeted delivery

Exosomes are naturally secreted, extracellular vesicles (of diameter, 30–150 nm) derived from a wide range of cell types. Because of their intrinsic biocompatibility, low immunogenicity, natural targeting capacity, and ability to cross biological barriers (including the blood-brain barrier), exosomes have emerged as an attractive drug delivery system [[97], [98], [99]]. Functionally, these lipid bilayer-enclosed nanovesicles can be loaded with a range of bioactive cargoes, including proteins, lipids, and nucleic acids [100].

Clinically, exosome-based therapies are advancing rapidly, with notable progress in multiple fields, including oncology, where tumor-derived exosomes are utilized for antigen presentation in cancer vaccines; neurology, where MSC-derived exosomes are being exploited to treat neurodegenerative diseases; and immunotherapy, where exosome-mediated PD-1/PD-L1 blockade is being explored to enhance antitumor immune responses [[101], [102], [103], [104]]. Because the surface membrane proteins of exosomes are derived from the parent cell, most native exosomes also demonstrate tissue tropism (as an example, integrins on the surface are responsible for organotropic targeting). This tropism can be further enhanced via surface engineering approaches, including antibody conjugation or viral glycoprotein pseudotyping.

Despite the considerable advantages of exosomes, challenges persist in their isolation and purification, yield limitations, and heterogeneity. In addition, a considerable proportion of exosomes is cleared by the mononuclear phagocyte system [[105], [106], [107]]. To address this limitation, strategies such as surface PEGylation and CD47 signal incorporation have been adopted to prolong systemic circulation [108]. Nonetheless, several recent advances have underscored the translational potential of exosomes: inhalable exosome-based SARS-CoV-2 vaccines have been shown to induce mucosal immunity; and stem cell-derived exosomes have proven regenerative efficacy in preclinical models of myocardial infarction [109]. Together, these findings support the broad utility of exosomes across diverse therapeutic domains.

### 3.5 Cell-penetrating peptides: fundamental mechanisms and expanding therapeutic application

Cell-penetrating peptides (CPPs) are short peptide fragments (typically 10–30 amino acids) with cationic or amphipathic properties that enable them to cross cellular membranes and deliver diverse molecular cargoes [110,111]. Since the discovery of HIV-1 Tat peptide in 1988, representative CPPs—including Tat, penetratin, transportan, and polyarginine—have been extensively exploited in non-viral delivery vectors for nucleic acids, proteins, nanoparticles, and small molecules [112]. CPPs are internalized via direct membrane translocation and endocytosis, and they can be chemically conjugated or non-covalently complexed with cargoes [113,114]. More recently, CPPs have been repurposed as non-viral delivery platforms for CRISPR/Cas9 ribonucleoproteins; for example, a PepFect14-based CPP complex was shown to enable Cas9 RNP delivery at low nanomolar doses, thereby highlighting the promise of CPP-mediated RNP delivery for therapeutic genome editing [115].

Clinically, CPPs have entered clinical investigation primarily as anticancer agents and as tumor-targeting payload carriers, with the 28-amino-acid peptide p28 advancing through first-in-human studies that demonstrated its safety and pharmacokinetics in adult and pediatric patients with advanced solid tumors [116]. While clinical translation beyond p28 remains limited, expanding preclinical programs that use CPPs to deliver chemotherapeutics, protein therapeutics, imaging agents, and oligonucleotides, have been detailed in recent reviews [117]. In research developing therapeutics for the central nervous system, CPP fusions have shown high neuroprotective activity in animal stroke models, and they are also being explored as carriers for neurotrophic factors and other biologics [118].

In recent work, prebinding or grafting blood–brain barrier (BBB) shuttle peptides or CPPs such as LAH4 and THR onto AAV capsids was shown to enhance brain transduction after systemic administration, substantially increasing CNS gene delivery while reducing the doses required [119]. After engineering AAV9 capsid variants that display cell-penetrating peptides, Yao and colleagues demonstrated markedly improved BBB penetration and widespread central nervous system transduction in both rodents and nonhuman primates [120]. Hołubowicz and colleagues reported that the incorporation of cell-penetrating peptides into optimized lipid nanoparticle formulations markedly enhanced the delivery of base-editing and prime-editing ribonucleoproteins. Combined LNP-CPP systems achieved a greater than three-hundred-fold increase in *in vivo* gene-editing efficiency with minimal off-target effects (compared with naked RNPs), demonstrating a safer and more effective strategy for non-viral genome editing [121]. However, cytosolic bioavailability of CPP cargoes is often reduced by endosomal entrapment (unless endosomal escape is specifically engineered), undermining functional activity [122]. Furthermore, proteolytic instability and rapid renal clearance can limit systemic exposure, necessitating chemical modifications that improve serum stability. However, these modifications can come at the cost of reduced uptake or increased toxicity. In addition, off-target uptake and lack of cell type specificity can create safety and dosing challenges for systemic applications; indeed, immunogenicity and unexpected toxicities have already been observed in some studies, complicating clinical development [117]. Finally, scalable manufacturing, reproducible formulation, and regulatory pathways for peptide-based delivery systems remain practical hurdles that must also be addressed [116]. Cell-penetrating peptides (CPPs) will maintain a role as flexible tools for intracellular delivery in research settings and in localized therapeutic applications. Their ability to cross cellular membranes continues to make them attractive for mechanistic studies and modular design. However, their use in systemic *in vivo* gene or protein delivery remains difficult. CPPs are rapidly degraded in biological fluids, show limited tissue selectivity, and are cleared quickly from circulation. These features reduce their effective dose and increase the risk of off-target uptake. In addition, several CPPs exhibit dose-related cytotoxicity, which further limits their clinical potential. As a result, CPPs are more suited to collaborative workflows where they complement other delivery systems, rather than serving as independent platforms for broad clinical translation.

### 3.6 Gold nanoparticles (AuNPs): structurally tunable inorganic carriers for gene therapy

Gold nanoparticles (AuNPs) are spherical inorganic particles (typically, 5–50 nm in size) that are valued for their favorable physicochemical properties, intrinsic biocompatibility, and exceptionally tunable surface chemistry [123,124]. The gold core provides a robust platform for strong thiol-gold chemistry that enables covalent attachment of oligonucleotides, peptides, proteins, and small molecules; permitting the construction of multifunctional conjugates including spherical nucleic acids and Cas9 ribonucleoprotein carriers [125,126]. To date, AuNPs have been used to deliver a broad spectrum of therapeutic cargoes, from siRNAs and mRNAs to CRISPR–Cas9 ribonucleoproteins and small-molecule drugs; moreover, functional delivery of these cargoes has been demonstrated both in vitro and *in vivo* when the AuNPs are formulated with endosomal-disruptive polymers or cationic coatings [127]. Surface functionalization of AuNPs with polyethylene glycol (or related hydrophilic polymers) markedly improves colloidal stability in biological fluids, reducing the adsorption of serum proteins and opsonins, and prolonging systemic circulation; however, PEGylation can also alter cellular uptake and must be optimized for each application [128]. Exploiting these features, proof-of-principle systems—such as CRISPR-Gold, in which 15 nm gold cores coated with thiolated DNA were complexed with Cas9 RNP and an endosomal-disruptive polymer—have achieved *in vivo* homology-directed repair and functional correction of disease mutations in mouse models, demonstrating the translational potential of AuNP platforms for gene editing and nucleic acid therapy [129].

AuNP delivery efficiency is highly size-dependent, with particles in the 15–20 nm range demonstrating optimal cellular uptake via clathrin-mediated endocytosis [130]. Targeting specificity can be governed by incorporating various surface ligands: folate enables selective delivery to FRα-positive tumors; RGD peptides target ανβ3 integrins; and antibody conjugates provide disease-specific recognition [131]. Notably, folate-conjugated AuNPs have been developed as an effective targeted nanoplatform for cancer therapy, and these have been shown to demonstrate enhanced cellular uptake in folate receptor-positive tumors and superior therapeutic efficacy compared with non-targeted systems [132]. A Phase 0 clinical study of RNA interference-based spherical nucleic acids (NU-0129) in recurrent glioblastoma provided the first evidence of clinical safety and tumor-specific delivery of AuNP-based gene therapeutics, highlighting their translational potential [133]. Beyond cancer therapy, AuNP-based systems have also been applied in neurological disorders. For example, AuNP-based delivery of CRISPR into fragile X syndrome mouse model brain rescued mice from exaggerated repetitive behaviors, demonstrating that AuNP-mediated gene editing can achieve functional neurological recovery *in vivo* [134].

Despite these advances, AuNPs face clear barriers to broad clinical translation for systemic gene or protein delivery. Their biodistribution favors liver and spleen accumulation, which limits delivery to many target organs and raises concerns about long-term accumulation and toxicity [135]. Endosomal entrapment of AuNPs also remains an obstacle [136]. Strategies like photothermal-triggered release via near-infrared (NIR) laser irradiation have enabled “on-demand” endosomal escape and subsequent siRNA release into the cytoplasm [137]. However, challenges persist, including: variability in large-scale synthesis and the necessity for precise spatiotemporal control of cargo release [138]. Innovations such as hybrid platforms that combine AuNPs with extracellular vesicles and the use of pH- or enzyme-cleavable linkers are being explored to address these limitations. These strategies support the development of AuNP-based systems as a flexible platform for next-generation gene therapies and theranostic applications. Additionally, AuNPs will keep a strong niche advantage in applications that exploit their unique optical and surface properties, such as local photothermal treatment and multifunctional theranostics [139].

### 3.7 Mesoporous silica nanoparticles (MSNs): high-capacity modular nanocarriers with translational challenges

Mesoporous silica nanoparticles (MSNs) in the 50–200 nm size range feature ordered mesopores (2–50 nm) and exceptionally high surface areas (>500 m^2^ g^−1^). Their large internal pore volumes enable high cargo-loading capacities (up to ∼30% w/w), and their chemical robustness, biodegradability, and versatile functionalization make them well-suited for a broad spectrum of therapeutic applications [[140], [141], [142]]. During in vitro studies, MSN uptake has been confirmed via clathrin-mediated endocytosis, and significant protection of encapsulated cargoes from enzymatic degradation has been demonstrated [142,143].

The translational challenges for future applications of MSNs include: scalable GMP-compliant production; comprehensive long-term biodistribution and toxicity assessments; and precise tuning of biodegradation kinetics to balance therapeutic persistence with safety. Furthermore, *in vivo* delivery of MSNs remains a challenge because of their low targeting specificity and rapid clearance via hepatic and splenic uptake [144]. Silica accumulation and toxicity concerns have also been highlighted in multiple preclinical studies. Emerging formulation strategies address these limitations by incorporating polyethylene glycol (PEG) to prolong circulation, functional polymers to facilitate endosomal escape, and zwitterionic surface coatings to mitigate protein corona formation. Targeting strategies for MSNs have explored both passive accumulation via the enhanced permeability and retention effect, and the active surface conjugation of targeting ligands to increase tissue specificity. A systematic comparison of non-viral vector delivery systems is provided in Table 3.

## 4 New emerging delivery systems

Delivery vehicles for gene therapy are not necessarily limited to traditional tools. While viral and non-viral vectors are widely used, these delivery platforms still have significant limitations. To circumvent these issues, new delivery tools are being explored, and several with potential are already being developed. For example, homologs of virally encapsulated structures and novel structures with phage-injection-like functionality (such as PVC and T6SS) are currently being scrutinized for their ability to package nucleic acids or proteins for delivery.

The most promising tools are likely to be based on retrovirus-like structures of different species origin that share a common evolutionary ancestor with Ty3-gypsy LTR retrotransposons, an evolutionary close relative of HERV. The three sets of core genes (gag, pol, and env) encoded by retroviruses each produce multiprotein precursors. The Gag encoded proteins constitute the viral core particles that contain structural domains required for viral assembly and release. The matrix (MA) structural domain guides Gag to the plasma membrane and facilitates binding of the viral envelope (Env) glycoprotein. The capsid (CA) structural domain drives Gag-Gag interactions during assembly, while the nucleocapsid (NC) structural domain encapsulates the viral genomic RNA. The pol gene encodes reverse transcriptase, RNase H, and integrase activities. Lastly, the env gene encodes proteins that are embedded in the viral envelope (which is derived from the host cell membrane), and is responsible for mediating target cell infection [145].

**Fig. 4:**
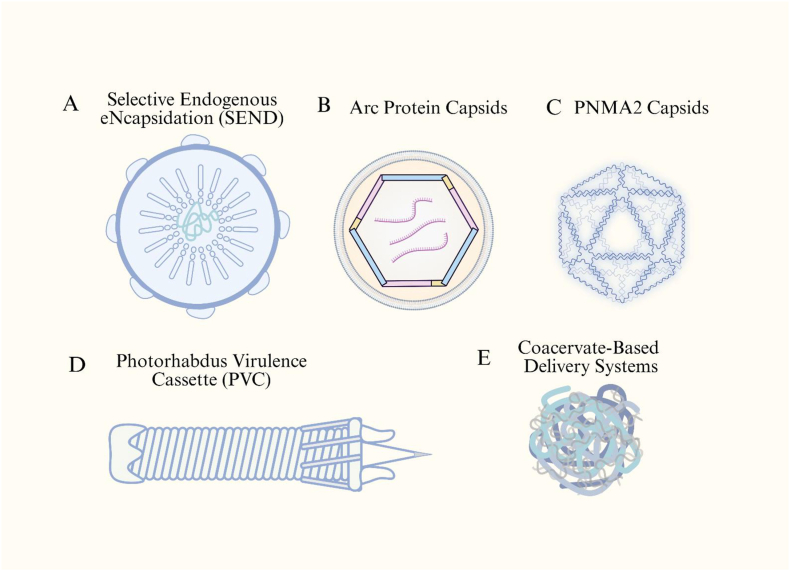
**New emerging delivery systems.** Schematic overview of next-generation non-viral delivery platforms inspired by synthetic and natural biological assemblies. These include (A) the Selective Endogenous eNcapsidation for cellular Delivery (SEND) system, which leverages endogenous retroelement-derived PEG10 proteins to package and transfer RNA; (B) Arc protein capsids originating from neuronal retrotransposon homologs that mediate intercellular mRNA transfer; (C) PNMA2 capsids, structurally related to retroviral Gag proteins with potential for self-assembly–based delivery; (D) Photorhabdus virulence cassettes (PVCs), contractile injection systems capable of protein translocation; and (E) coacervate-based delivery systems, which employ membraneless phase-separated droplets for the encapsulation and cytosolic release of proteins or nucleic acids. Collectively, these bioinspired systems expand the landscape of programmable and biocompatible vectors beyond conventional viral and lipid-based platforms. Created in https://BioRender.com.

Gag is the main structural protein of the package, and its functions include preventing the cargo from being degraded by enzymes. This is typically the main obstacle in the delivery process. Hence, structural and functional characterization of the Ty3-gypsy type LTR retrotransposon has led to the potential development of novel delivery vehicles. Fig. 4 summarizes the structural of new emerging delivery systems.

### 4.1 Arc capsids: an endogenous neuronal messenger reimagined for therapeutic delivery

Arc (Activity-regulated cytoskeleton-associated) protein is a human endogenous retrovirus-like protein expressed predominantly in neurons [146,147]. The neuronal *Arc* gene originated from the Ty3 retrotransposon, derived from a retrotransposon *Gag* gene that has been fixed in the mammalian genome and is therefore a fully endogenous protein. During development, central or peripheral immune tolerance to Arc is established, suggesting that Arc is less likely to elicit strong immune recognition compared with exogenous viral proteins [148]. Arc has been shown to form a bilayer capsid of approximately 20–60 nm, and to contain structures found in viral group-specific antigen (Gag) polyproteins, including the MA/CA structural domains of the retroviral Gag-like protein (but not the NC domain); while the architecture of dArc1 CA is highly related to that of orthoretroviral and spumaretroviral structures, the amino-terminal and carboxyl-terminal domains are substantially different [148,149]. Arc capsids have neuronal mRNA packaging capabilities, and the capacity to self-assemble into virus-like capsids that encapsulate RNA. Interestingly, endogenous Arc protein is released from neurons in extracellular vesicles that mediate the transfer of Arc mRNA into new target cells [148]. Kritika Mehta et al. previously demonstrated that phosphatidylinositol-3-phosphate (PI3P) mediates Arc capsid assembly and secretion through the endosomal-multivesicular body (MVB) pathway [150]. However, applications of native Arc capsids are limited by their selective packaging of endogenous neuronal mRNAs, and the absence of cell-type targeting specificity. Moreover, their intrinsic delivery efficiency remains suboptimal (when compared with conventional viral or nonviral vectors) because specific RNA encapsidation does not occur.

Despite these limitations, Arc vesicles offer significant advantages, including their endogenous origin, neural specificity, and inherently low immunogenicity. Rationally engineered Arc capsids hold promise as biocompatible, neuron-targeted delivery platforms capable of trans-synaptic transport, and they may even be capable of traversing the BBB. Vaibhav Upadhayay et al. reported that addition of the HIV-1 NC domain to the C-terminus of rArc significantly improved its binding to HIV-1 5′ UTR [151]. Li et al. reported that by exploiting the capsid-like assembly of Arc, the nanoparticles enabled multivalent antigen presentation and induced effective antigen-specific immune responses in mice with relatively low anti-scaffold immune responses. These findings suggest Arc as a biologically derived and engineerable delivery scaffold [152]. Anticipated advances include hybrid Arc–lipid nanoparticle constructs to enhance systemic stability, and RNA-guided encapsulation systems enabling the loading of diverse RNA therapeutics or small peptide cargoes. In future applications, Arc-derived vectors may facilitate the precise delivery of CRISPR-based genome editors or functional proteins into selected neuronal subtypes, thereby unlocking new therapeutic avenues for neurodegenerative and neuropsychiatric diseases.

### 4.2 SEND: an RNA packaging system (PEG10-Based delivery platform) enabling practical delivery

The SEND (Selective Endogenous eNcapsiation) platform is a relatively mature delivery system for producing PEG10-derived particles that selectively encapsulate RNA cargoes containing PEG10 binding sequences, thereby enabling localized RNA delivery [153]. PEG10 protein includes the CA/NC domains and parts of POL, including PRO and RT, and it has the unique ability to self-assemble into virus-like particles (VLPs). PEG10 protein naturally forms ∼100 nm enveloped VLPs through ESCRT-mediated budding, and it exhibits several unique molecular characteristics, including RNA-binding domains that specifically package mRNA or CRISPR guide RNAs, and high humanization [153,154].

Its exciting potential notwithstanding, SEND remains at an early stage of development (when compared to established viral and non-viral vectors), and its gene transfer efficiency remains approximately four-to five fold lower than that achieved with integrating lentiviral vectors [155]. Nonetheless, efficient in vitro delivery of functional mRNAs and CRISPR–Cas components has already been demonstrated, with the SEND system achieving targeted gene editing in human and murine cells of up to 40% and 30%, respectively [153]. Additionally, functional protein expression was achieved through endocytic uptake or VSV-G-mediated membrane fusion. Although PEG10-based systems have yet to achieve a clinical application, *in vivo* applications of SEND-derived particles have been explored in preclinical investigations, including for ocular mRNA delivery and for immune modulation via engineered PEG10 VLPs [156,157]. The SEND platform is now being actively advanced by biotechnology companies such as Aera Therapeutics. To address the key challenges of biodistribution, immunogenicity, and scalable manufacturing, these companies are advancing the optimization of PEG10 variants, fusogen pseudotyping, and the development of lyophilized formulations.

### 4.3 PNMA2 capsids: designer retrotransposon shells for next-generation mRNA encapsulation

Paraneoplastic antigen MA2 (PNMA2) is a Gag homolog derived from the Ty3 retrotransposon [158]. PNMA2 is associated with paraneoplastic neurological syndrome and is predominantly expressed in the brain, although it is also expressed in paraneoplastic tumors from other tissues [159]. In comparison with Gag, PNMA2 only includes CA/NC (and not MA), and it too forms virus-like capsids that are released from cells [160]. As outlined below, PNMA2 particles engineered with RNA-binding pockets facilitate efficient mRNA encapsulation and reduced immunogenicity, and they may soon be exploited as a delivery system.

The structure of the PNMA2 capsid was resolved by Feng Zhang and colleagues using cryo-EM. The native structure of the PNMA2 capsid has a diameter of approximately 200 Å (20 nm; around 60% that of AAV-2), and it is specifically composed of 12 pentameric capsid grains and 60 individual mPNMA2 molecules [160]. Although purified PNMA2 protein spontaneously forms ordered virus-like capsids, it does not package its own mRNA (or any cellular mRNA), expressly because the interior of the capsid has a negative charge. This deficit can be reversed by replacing the C-terminal disordered region of PNMA2 with an RNA-binding motif derived from the N-terminal 30 residues (CCMV1–30) of cowpea chlorotic mottle virus. Additionally, LAH4 aid proteins can be incorporated into the system to enhance cellular entry and endosomal escape [160]. In their milestone study, Feng Zhang and colleagues developed engineered PNMA2 as an emerging non-viral mRNA delivery platform, packaged mRNA molecules into the icosahedral capsids using recombinant and purified ePNMA2 protein, and demonstrated its usefulness as a delivery agent for mammalian cell lines [160].

Currently, development efforts are focused on preclinical animal testing (biodistribution, dosing, immune safety), epitope engineering to reduce immunogenicity, optimization of packaging and capsid yield, and surface engineering for targeting or fusion. If successful, ePNMA2 could potentially become a complementary modality to lipid nanoparticles and engineered viral vectors, particularly for indications requiring repeated dosing and low immunogenicity. Table 5 summarizes representative emerging delivery systems beyond conventional viral vectors.

**Table 5:** New emerging delivery systems with translational potential.

	PNMA2 (paraneoplastic antigen Ma2)	SEND (Selective Endogenous eNcapsiation)	PVC (Photorhabdus Virulence Cassette)	ARC (Activity-regulated cytoskeleton-associated)	Coacervate based delivery systems
Molecule	Endogenous Gag-like protein; forms non-enveloped icosahedral capsids when expressed.	PEG10 is an endogenous retrotransposon-derived Gag homolog that forms VLP-like particles when expressed, natural MA domain deletion	Bacterial Contractile Injection System (Class I Extracellular Contractile Injection System eCIS), Natural macromolecular complexes	Self-assembly into VLP structures, natural absence of the NC (nucleocapsid) domain	Coacervate-based transfection reagent (commercial formulations such as “ProteanFect”); typically positively charged polymers forming nanoparticles via electrostatic interaction with nucleic acids.
Delivery contents	mRNA、siRNA	mRNA	Cas9、RNP、ssDNA	Neuronal mRNA	mRNA、siRNA and DNA (pDNA)
Packaging capacity	Lack of natural packaging capability (unable to incorporate RNA), it can encapsulate RNA through electrostatic interactions or spatial structure of the fusion protein after modification	Moderate, capable of efficiently packaging linear RNA <5 kb. MmPEG10 can bind and secrete its own mRNA, and successfully delivers 5 kb SpCas9 mRNA	Capable of packaging 170 kDa Cas9 protein, as well as loading various macromolecules such as RNP and ssDNA.	Natural prioritization of packaging endogenous mRNA, due to NC deficiency, exhibits the characteristic of actively packaging non-target RNA.	Loading is achieved through electrostatic binding between cationic groups and the negatively charged backbone of nucleic acids. The loading capacity is regulated by adjusting the polymer-to-nucleic acid ratio.
Modifiability	Medium, C-terminal fusion virus capsid proteins (e.g., CCMV) that perform RNA packaging functions	High, modular platform, addition of fusogens or targeting elements in SEND designs.	High, by editing the target recognition domain of tail fibrinogen Pvc13 or conjugating antibodies/ligands, enables precise targeting of specific cells.	Unknown	Moderate, by modifying PEG, incorporating targeted ligands (such as peptides or antibodies), or optimizing the polymer backbone structure, to enhance biocompatibility, targeting efficiency, and nucleic acid loading/release performance.
Delivery efficiency	Forms non-enveloped, self-assembling capsids; however, PNMA2 particles are not reported to naturally encapsidate nucleic-acid cargos.	enable endogenous encapsidation of RNA and can mediate intercellular transfer; reported intracellular delivery/release is promising but highly dependent on cargo design and cellular context	The membrane-penetrating agent directly traverses cell membranes via a natural contraction injection mechanism.	Released through EVs	High membrane permeability, good intracellular release efficiency
Targeting	Originates from neurons and shows preferential uptake in neural/immune contexts, but lacks a defined specific receptor-mediated targeting mechanism; targeted delivery would require surface engineering or ligand conjugation.	Intrinsic targeting specificity is limited; SEND systems are amenable to modular engineering to achieve cell-type selectivity.	Retargeting by engineering recognition domains (e.g., Pvc13) is a plausible strategy demonstrated in some models.	Tropism for neuronal circuits and synaptic compartments in reported studies.	Unmodified coacervates typically lack intrinsic cell-type specificity.
In vitro and *in vivo* stability	High stability, high concentrations of detergent do not damage the structure.	In vitro VLP-like assemblies show moderate structural stability and may be protease-sensitive; being derived from human proteins could reduce immunogenic clearance *in vivo*, but this depends on expression level, modification and host immune status.	Moderate stability outside the body; suitable for long-term storage at −80 °C.	Sensitivity to ionic conditions, similar to HIV capsids	Require formulation strategies for improved circulation stability.
Safety	Highly immunogenic	Low, fully endogenous system, assembled from fully humanized PEG10 protein.	Native bacterial components are likely immunogenic.	Low, endogenously self-assembled protein capsid	Biocompatibility depends heavily on polymer chemistry and formulation; some coacervate materials are well tolerated
Advantages	Self-assembling capsid-forming protein with potential as an engineered display or delivery scaffold; however, PNMA2 particles are not reported to naturally encapsidate nucleic acids — cargo loading would require deliberate engineering	Fully humanized design results in extremely low immunogenicity and excellent biocompatibility. The modular platform allows flexible modification and enables efficient delivery of linear RNA, payload size limits and in-vivo performance remain under active investigation	High delivery efficiency (direct transmembrane penetration + efficient release), strong targeting capability (precision targeting achievable via Pvc13 modification), robust packaging capacity.	Arc forms capsid-like particles capable of mediating intercellular RNA transfer in neuronal circuits; promising for neural-targeted applications.	Coacervates can effectively concentrate nucleic acids and facilitate cellular uptake, showing high transfection in some primary and hard-to-transfect cells.
Challenges	Natural MA cannot autonomously package RNA in human cells and requires C-terminal modification (e.g., CCMV fusion); expression and purification in animal cells are complex	Natural MA deficiency, production dependent on animal cells with complex purification, long-term safety unknown.	Immunogenicity of bacterial proteins	Natural NC deletion leads to active packaging of non-target RNA, with limited modifiability.	Low natural targeting efficiency and high concentration pose cytotoxic risks.

### 4.4 PVC: A bacterial nanosyringe repurposed for precision protein delivery

The Photorhabdus Virulence Cassette (PVC) system is a naturally occurring microbial nanomachine with groundbreaking potential for development as a precision protein delivery system. PVC is a member of the bacterial extracellular contractile injection systems (eCISs) class, which has evolved to puncture target cell membranes and then inject payload proteins directly into host cells [161,162]. Similar to other eCISs, PVC is structurally reminiscent of phage tail “nano-syringes” and is ∼100 nm in length. It also has the ability to self-assembly, and the capacity to deliver large therapeutic proteins, such as Cas9 nucleases. For example, human-targeting PVCs can deliver SpCas9 protein into HEK 293FT cells and induce indels at a rate of 10∼15% [162]. PVC targeting is achieved via engineered Pvc13 tail fiber proteins, which can be reprogrammed to recognize human cell surface markers like EGFR. Mechanistic studies have confirmed that Pvc15, an AAA ATPase with tandem D1/D2 domains, is essential for efficient payload loading; thus, truncations or mutations in Pvc15 compromise loading [163]. Additionally, N-terminal disordered “signal peptides” (SPs) must be integrated into the payloads for recognition and incorporation into the PVC tube lumen. Hence, the payload can be changed to realize differential delivery of different contents.

While PVC delivery systems show exceptional promise, especially for cancer therapy and biocontrol, several key challenges remain. Most notably, immunogenicity modulation is required because the PVC system is of bacterial origin; if homologs of human origin can be found, the clinical applications of PVC would be greatly broadened [162,164]. PVC delivery systems also require a protocol that can be prepared on a large scale, facilitating fast progression from the laboratory stage to the clinic. Current clinical advances are focused on pipeline development, with Aera Therapeutics playing a prominent role. The main aim is to explore PVC variants for *in vivo* CRISPR delivery and chimeric antigen receptor (CAR) protein therapies. This platform's unique capacity for cytosolic delivery has positioned it as a transformative alternative to viral vectors and LNPs, pending resolution of long-term safety and manufacturing hurdles.

### 4.5 Coacervate-based delivery systems: phase-separated soft carriers bridging biomolecular condensates and gene therapy

Coacervate-based delivery systems, formed by liquid–liquid phase separation (LLPS), are a rapidly emerging class of biomimetic carriers with unique physicochemical and biological properties. These systems are formed through spontaneous electrostatic complexation between oppositely charged macromolecules—such as proteins, peptides, nucleic acids, or synthetic polyelectrolytes—and they generate membrane-less, liquid-like droplets known as coacervates. This process closely resembles the natural formation of biomolecular condensates such as stress granules and P-bodies within cells, providing an attractive platform for the controlled encapsulation and intracellular delivery of biological cargoes [165,166].

A key advantage of coacervate systems lies in their high loading capacity for diverse therapeutics, including nucleic acids, proteins, enzymes, and small molecules, which is achieved through multivalent electrostatic and hydrophobic interactions [167]. Moreover, the assembly process is generally mild and aqueous, avoiding organic solvents, surfactants, or high shear stress, thereby preserving the structural integrity and biological activity of sensitive cargoes. Despite these advantages, coacervate-based systems still face several challenges that limit their clinical translation. The complex and multivariate nature of phase separation makes reproducible preparation difficult, as small variations in charge density, polymer length, or environmental conditions can drastically alter droplet formation [168]. Additionally, large-scale production of coacervates with consistent physicochemical properties requires refined process engineering and standardization [169].

**Fig. 5:**
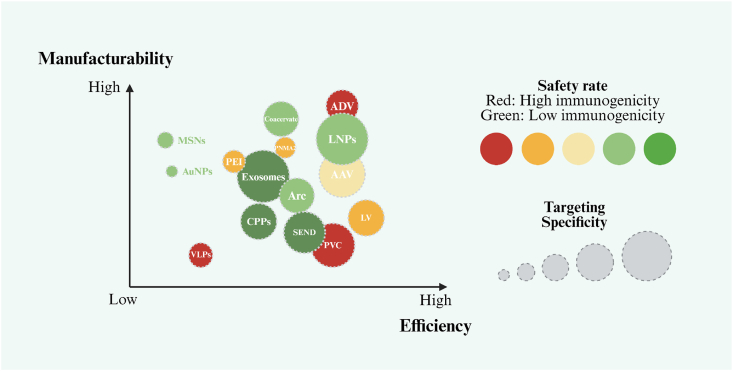
Comparison of representative viral and non-viral delivery platforms in terms of manufacturability, delivery efficiency, immunogenicity, and targeting specificity. The x-axis represents delivery efficiency, while the y-axis denotes manufacturability. Bubble color reflects immunogenicity level (red indicates high immunogenicity, green indicates low immunogenicity). Bubble size corresponds to targeting specificity. Compared with viral vectors (AAV, ADV, LV), non-viral systems such as LNPs, PEI, MSNs, AuNPs, CPPs, VLPs and exosomes exhibit superior manufacturability and lower immunogenicity, though their delivery efficiency and targeting ability vary across platforms. Created in https://BioRender.com.

In recent years, the applications of coacervate delivery systems have expanded across multiple biomedical areas. In gene delivery applications, coacervates formed by arginine-rich proteins and RNA have achieved high transfection efficiency in hard-to-transfect primary and immune cells, outperforming lipid-based reagents. Protein–RNA coacervates such as the ProteanFect/PhaseX platform (also known as the “EASY platform”) demonstrate efficient delivery of mRNA, siRNA and pDNA. By leveraging a mammalian endogenous protein that forms coacervates with nucleic acids through LLPS, EASY achieves efficient cytoplasmic release and broad compatibility across diverse primary cell types, including human T cells, NK cells, and hematopoietic stem cells. Functionally, EASY demonstrates exceptional versatility, achieving >90% mRNA transfection efficiency while maintaining high cell viability; moreover, it supports multiplex genome editing. The EASY platform facilitates precise genomic integration via homology-directed repair (HDR), enabling insertion of therapeutic constructs such as anti-CD19 CAR sequences into endogenous loci to enhance CAR-T performance. The robust and non-viral nature of EASY eliminates risks associated with insertional mutagenesis and immune activation, positioning it as a promising next-generation alternative to both viral and LNP-based delivery systems. Collectively, this coacervate-driven strategy represents a conceptual leap in gene therapy, bridging biomolecular self-assembly principles with clinical gene editing applications, potentially redefining scalable, safe, and high-efficiency delivery for engineered cell therapies. Fig. 5 reflects an author-informed qualitative estimation rather than an absolute evaluation.

## 5 Discussion

Over the past decade, the rapid advancement of gene editing technologies has profoundly transformed biomedical research, offering unprecedented opportunities for understanding life processes and for developing transformative therapies. However, clinical translation of these new therapies remains constrained by the limitations of the available delivery systems. Indeed, efficient, precise, and safe delivery vehicles are the cornerstone for bridging laboratory discoveries with real-world applications.

In this review, we have systematically summarized the major gene delivery platforms, with an account of conventional non-viral systems (including lipid nanoparticles, polyethyleneimine, gold nanoparticles, mesoporous silica nanoparticles, and cell-penetrating peptides), several widely used viral vectors (including adenovirus, adeno-associated virus, lentivirus, and virus-like particles), and the emerging bio-inspired platforms (including the PEG10-based SEND system, PNMA2 protein capsids, the bacterial *Photorhabdus* virulence cassette system, Arc protein capsids, and coacervate-based condensates). Notably, many of these novel bio-inspired platforms represent new paradigms that combine programmable structures with intrinsic biocompatibility. The emergence of these novel delivery systems points toward a clear direction for future platform development, namely the exploration of human-derived proteins as delivery scaffolds. In drug development, humanization remains one of the most established and effective strategies for reducing immunogenicity. Aligning candidate sequences with the human genome to maximize sequence similarity can substantially mitigate immune recognition. From this perspective, fully human-derived delivery systems represent an ideal design goal and offer an important framework for the development of next-generation biocompatible delivery platforms. Leveraging delivery tools based on novel principles, next-generation delivery technologies are expected to incorporate spatiotemporal control mechanisms such as ultrasound responsive delivery systems to achieve more precise and on-demand cargo release [170]. In parallel, advances in fully biodegradable polymeric vectors, including CO_2_-derived polycarbonates, further illustrate the growing emphasis on safety-conscious and environmentally benign design principles in next-generation delivery systems [171,172]. Together, these innovations underscore a clear trend toward designing gene delivery platforms that are highly effective and inherently safe.

While non-viral vectors offer tunable chemistry, scalable production, and low immunogenicity, they still face significant challenges, especially concerning *in vivo* efficiency, endosomal escape, and tissue targeting. Conversely, viral vectors can achieve high transfection efficiency and stable gene expression. However, their challenges include fixed tropism, limited cargo capacity, and elevated immunogenicity. Future developments in delivery systems will emphasize biocompatibility, modularity, and programmability. The integration of AI-assisted molecular design with synthetic biology approaches may enable next-generation bio-inspired carriers that synergize the advantages of both viral and non-viral systems. Ultimately, the ideal delivery platform should combine high safety, precise specificity, and scalable manufacturability, to enable efficient delivery of nucleic acids, proteins, and hybrid biomolecules. As these technologies mature, delivery innovation will advance gene therapy and precision medicine, and thereby reshape biotechnology across agriculture, environmental science, and synthetic biology.

## CRediT authorship contribution statement

**Hengyi Wang:** Writing – original draft, Visualization. **Xiaoyan Tang:** Writing – original draft, Visualization, Software, Formal analysis. **Xinyao Pan:** Writing – review & editing, Supervision, Formal analysis. **Hongjie Tang:** Supervision, Software, Resources. **Jie Gao:** Writing – review & editing, Supervision. **Qi Li:** Writing – review & editing, Writing – original draft, Visualization, Resources, Project administration, Funding acquisition, Conceptualization.

## Declaration of competing interest

The authors declare that they have no known competing financial interests or personal relationships that could have appeared to influence the work reported in this paper.
