# Supplementary material for: Advances in vehicles for in situ delivery: From classical vectors to biologically inspired structures
Source: Synth Syst Biotechnol. 2026 Mar 17;13:446–67. doi: 10.1016/j.synbio.2026.02.013 (PMC13011059; doi:10.1016/j.synbio.2026.02.013)
Supplement: Multimedia component 1 [file mmc1.pdf]

CERTIFICATE OF ENGLISH EDITING

This is to certify that the manuscript entitled

Advances in delivery systems: From classical vectors to biologically inspired structure.

commissioned to us has been carefully edited by a native English-speaking editor of MogoEdit, and the grammar, spelling, and punctuation have been verified and corrected where needed. Based on this review, we believe that the language in this paper meets academic journal requirements. Please contact us with any questions.

Gang Zhang

Dr. Gang Zhang
Founder & CEO of MogoEdit

Date of Issue
December 8, 2025

Disclaimer: Subsequent to our editing, a manuscript will be reviewed by the author(s) and then carefully rechecked by our editors during a second round of editing prior to submission. This manuscript however, received one round of editing only. The suggested edits in the document may therefore have been accepted or rejected by the authors at their sole discretion subsequent to our editing. Consequently, MogoEdit is not responsible for revisions made to the document after our last edit on **December 8, 2025.**

For authenticity verification, please scan the QR code provided. Simply open the camera app, point it at the code, and follow the link.

MogoEdit is a professional English editing company who provides English language editing, translation, and publication support services to individuals and corporate customers worldwide. As a company invested by the affiliate fund of Chinese Academy of Science, MogoEdit is one of the leading language editing service providers in China, whose clients come from more than 1000 universities and research institutes.
MogoEdit Website: http://www.mogoedit.com/
500+ native English editors: http://www.mogoedit.com/editors

make the world flatter

Mogo Internet Technology Co., LTD.
No. 57, 3rd Keji Road, Xi'an 710075, PR China +86 02988317483 support@mogoedit.com
